# Synthesis and crystallographic studies of 2-(di­phenyl­phosphino­thio­yl)-2-(3-oxobut-1-en-yl)ferrocene

**DOI:** 10.1107/S205698902100760X

**Published:** 2021-07-30

**Authors:** Uchchhal Bandyopadhyay, Basker Sundararaju, Rinaldo Poli, Eric Manoury, Jean-Claude Daran

**Affiliations:** a CNRS, LCC (Laboratoire de Chimie de Coordination), Université de Toulouse, UPS, INPT, 205 Route de Narbonne, F-31077 Toulouse Cedex 4, France; b Department of Chemistry, Indian Institute of Technology Kanpur, Uttar Pradesh, India

**Keywords:** crystal structure, organometallic chemistry, 2-(di­phenyl­phosphino­thio­yl)ferrocene chemistry, aldol/elimination reaction

## Abstract

As a follow-up to our research on the chemistry of disubstituted ferrocene derivatives, the synthesis and the structure of the title compound, 2-(di­phenyl­phosphino­thio­yl)-2-(3-oxobut-1-en-yl)ferrocene, [Fe(C_5_H_5_)(C_21_H_18_OPS)], are described. The mol­ecule is built up from a ferrocene unit disubstituted by an S-protected di­phenyl­phosphine group and by a methyl­vinyl­ketone chain. The crystal structure features weak C—H⋯O and C—H⋯S inter­actions, which build a two-dimensional network. This structure is compared to that of the related disubstituted di­phenyl­phosphino ferrocene.

## Abstract 2

The title mol­ecule is built up from a ferrocene unit disubstituted by an S-protected di­phenyl­phosphine group and by a methyl­vinyl­ketone chain. In the crystal, weak C—H⋯O and C—H⋯S inter­actions build a two-dimensional network.

## Chemical context   

Over the last few years, our team has developed several bidentate phosphine-containing planar chiral ferrocene ligands and tested them in various asymmetric catalytic reactions (Manoury & Poli, 2011 ▸). In particular, some P,O ligands were synthesized from 2-(di­phenyl­phosphino­thio­yl)ferro­cenecarboxaldehyde (Mateus *et al.*, 2006 ▸). This compound can be easily obtained as a racemic mixture or as each pure enanti­omer and bears a versatile aldehyde function, which can be used to obtain more complex mol­ecules. In this context, we were delighted to report a new and efficient aldol/elimination reaction of the aldehyde group to yield the corresponding ene-one under mild conditions (see Scheme) using a weak base (*p*Ka of 2-picolyl amine is 8.60; Miletti *et al.*, 2010 ▸). 
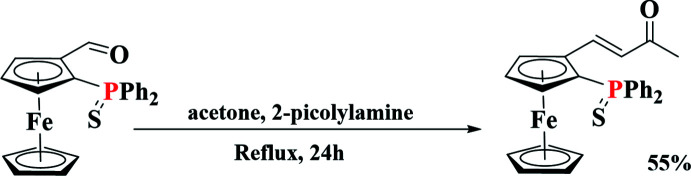


Similar compounds have been synthesized but using the Wittig reaction, which requires the synthesis of a specific phospho­nium reagent and the use of a strong base, such as *n*-butyl­lithium (Ye *et al.*, 2017 ▸; Schaarschmidt *et al.*, 2014 ▸; Štěpnička *et al.* 2008 ▸) or sodium hydride (Stepnicka *et al.*, 2008 ▸). Indeed, the aldol/elimination sequence has been used to functionalize ferrocenecarboxaldehyde, which is a much less crowded analog of 2-(di­phenyl­phosphino­thio­yl)ferro­cene­carboxaldehyde but with a much stronger base such as NaOH, KOH or tBuOK (see, for instance, Achelle *et al.*, 2012 ▸; Romanov *et al.*, 2015 ▸; Li *et al.*, 2020 ▸; Wieczorek *et al.*, 2016 ▸).

## Structural commentary   

**Figure 1:**
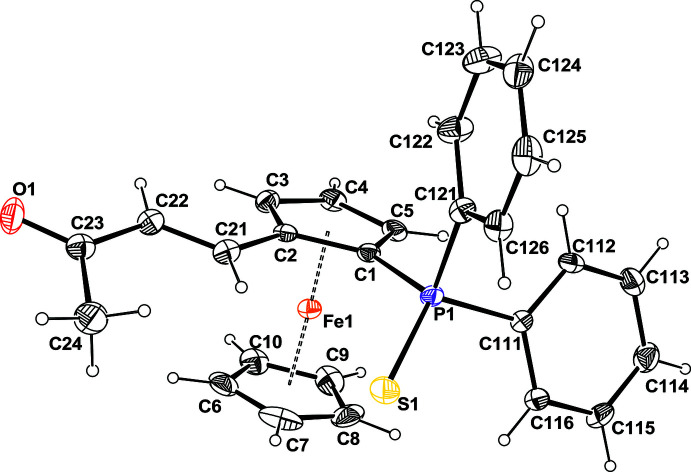
Mol­ecular structure of the title compound with the atom-labeling scheme. Ellipsoids are drawn at the 50% probability level and the H atoms are represented as small circle of arbitrary radii.

The mol­ecule is built up from a ferrocene unit disubstituted by an S-protected di­phenyl­phosphine group and by a methyl­vinyl­ketone chain (Fig. 1 ▸). As is usually observed for thio­phenyl­phosphine ferrocenyl derivatives, the P atom is roughly in the plane of the Cp ring, deviating from the mean plane by −0.034 (5) Å, whereas the S atom is offset from this plane by 1.159 (6) Å. The two Cp rings have a staggered conformation with a twist angle of *ca* 37.1°. The O atom is *trans* to the ferrocene unit with respect to the C=C double bond. The torsion angle of the C2—C21—C22—C23 chain is 172.4 (4)° and the plane containing the double bond is twisted with respect to the Cp ring by 22.8 (2)°. This mol­ecule has a planar chirality related to the occurrence of two different substituents on the Cp ring; however, as the space group is centrosymmetric, the two enanti­omers *R*/*S* are present in equal numbers within the crystal. Two intra­molecular C—H⋯S inter­actions occur (Table 1 ▸).

## Supra­molecular features   

**Figure 2:**
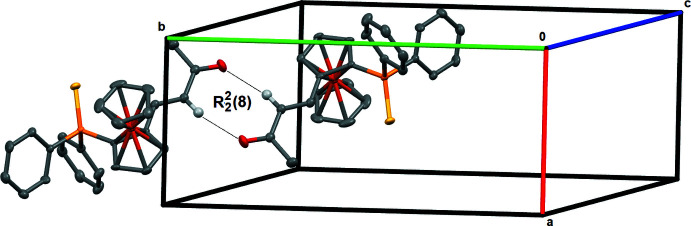
Partial packing view showing the formation of the 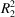 (8) pseudo-ring arranged around the (1/2, 1, 1/2) inversion center.

**Figure 3:**
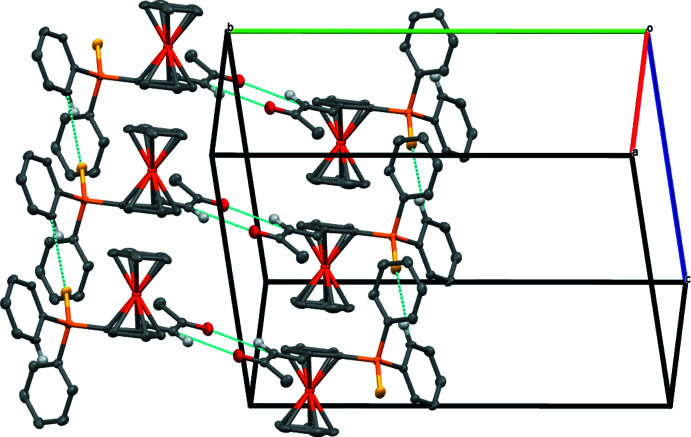
Partial packing view showing the formation of the ribbon parallel to the (0 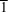 1) plane.

The packing of the structure is stabilized by weak C—H⋯O and C—H⋯S inter­actions (Table 1 ▸). The C—H⋯O inter­action results in the formation of a pseudo-dimer through an 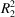 (8) graph-set motif (Etter *et al.*, 1990 ▸; Bernstein *et al.*, 1995 ▸) (Fig. 2 ▸). The C—H⋯S inter­cations build up a chain parallel to the *b* axis and these chains are further associated by the C—H⋯O inter­actions of the pseudo-dimer, building a ribbon parallel to the (0 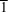 1) plane (Fig. 3 ▸).

## Database survey   

**Figure 4:**
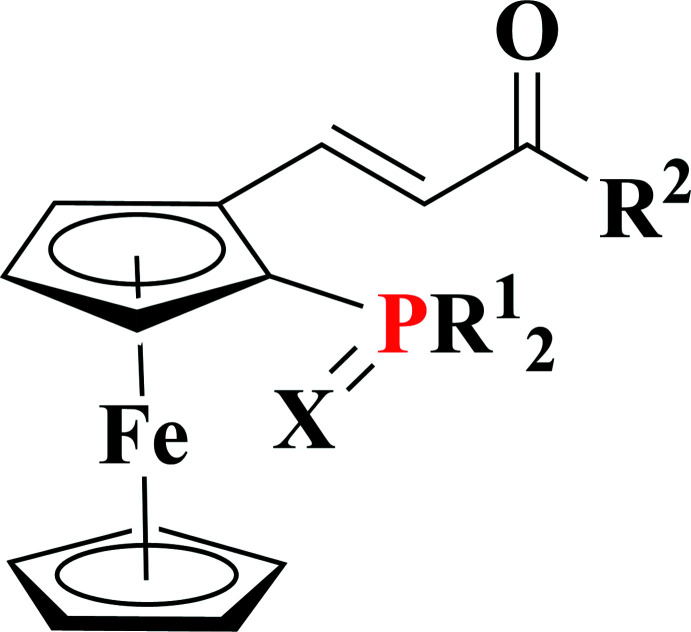
The model used for the CCDC search.

**Figure 5:**
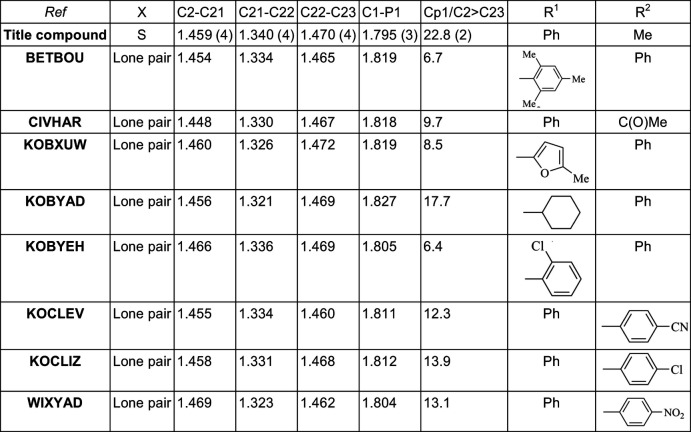
Comparison of bond distances (Å) and dihedral angles between the substituted Cp ring (Cp1) and the vinyl mean plane (°) for closely related compounds (CIVHAR: Stepnicka *et al.*, 2008 ▸; BETBOU: Kehr *et al.*, 2017 ▸; KOB***: Schaarschmidt *et al.*, 2014 ▸; WIXYAD: Iftime *et al.*, 2000 ▸).

A search of the Cambridge Structural Database (CSD version 5.42, update 2020.3; Groom *et al.*, 2016 ▸) does not reveal any structures with ferrocenyl disubstituted by a thiodi­phenyl­phosphine and a vinyl; however, a search using a fragment containing a ferrocenyl disubsituted by an unprotected phosphine and a vinyl substituent (Fig. 4 ▸) reveals 15 hits of which seven can be compared with the title compound, having only different substituents *R* ^1^ and *R* ^2^ (Fig. 4 ▸). A comparison of C—C and C—P distances and dihedral angles between the Cp ring and vinyl mean plane are shown in Fig. 5 ▸. Clearly the substit­uent on the phosphine has some influence on the C—P bond lengths, which range from 1.795 (3) Å for the title compound to 1.827 Å for the [η^5^-1-di­cyclo­hexyl­phosphino-2-(2-phenyl­ethen­yl)cyclo­penta­dien­yl](η^5^-cyclo­penta­dien­yl)iron com­pound (Schaarschmidt *et al.*, 2014 ▸) in which the phosphine bears two cyclo­hexyl substituents that are rather bulky. The occurrence of the S atom attached to the phosphine in the title compound may explain why the shortest value observed for the title compound. There is no significant difference in the C—C bonds within the vinyl moiety, showing that these values are not affected by the substituent, whereas the discrepancy observed for the dihedral angles between the vinyl unit and the Cp rings (6.4 to 22.8°) is related to the nature of the *R* ^1^ and *R* ^2^ substituents on the vinyl unit. The largest value of 22.8°, observed for the title compound, is related to the weak C21—H21⋯S1 inter­action.

## Synthesis and crystallization   

To a solution of 2-(di­phenyl­phosphino­thio­yl)ferrocene­carboxaldehyde (220 mg, 0.51 mmol) in acetone (40 mL) was added 2-picolyl­amine (0.2 mL, 1.53 mmol). The reaction mixture was refluxed for 24–36h with TLC monitoring of the consumption of aldehyde. After complete consumption, the reaction mixture was evaporated *in vacuo* and extracted with di­chloro­methane and washed with three portions of water. The combined organic layers were dried over Na_2_SO_4_, filtered and evaporated to dryness. The crude material was purified by silica gel column chromatography with a hexa­ne–ether mixture (1/1, *v*/*v*) to obtain the product as a red solid (0.13 g, 55%). Monocrystals suitable for X-ray diffraction analysis were obtained by slow diffusion of pentane into a di­chloro­methane solution of 4-(2-thiodi­phenyl­phosphinoferrocen­yl)-but-3-ene-one.

**^1^H NMR (ppm, CD_2_Cl_2_)**: δ 8.46 (1H, *d*, *J* = 16.3Hz, vin­yl); 7.90–7.80 (*m*, 1H, Ph); 7.65–7.15 (9H, *m*, Ph); 6.28 (1H, *d*, *J* = 16.3Hz, vin­yl); 5.01 (1H, *m*, subst. Cp); 4.65 (1H, *m*, subst. Cp); 4.39 (5H, *s*, subst. Cp); 4.07 (1H, *m*, subst. Cp); 3.87 (3H, *s*, CH_3_).

**^13^C NMR (ppm, CD_2_Cl_2_)**: δ 198. 16 (*s*, C=O); 143.46 (*s*, vin­yl); 134.93 (δ, *J*_CP_ = 87.4Hz, quat Ph); 133.01 (δ, *J*_CP_ = 86.6Hz, quat Ph); 132.03 (δ, *J*_CP_ = 11.0Hz, CH Ph); 131.69 (δ, *J*_CP_ = 10.7Hz, CH Ph); 131.54 (δ, *J*_CP_ = 3.0Hz, CH Ph *para*); 131.39 (δ, *J*_CP_ = 3.0Hz, CH Ph *para*); 128.40 (δ, *J*_CP_ = 12.5Hz, CH Ph); 128.19 (δ, *J*_CP_ = 12.4Hz, CH Ph); 126.89 (*s*, vin­yl); 83.06 (δ, *J*_CP_ = 10.7Hz, quat Cp); 77.44 (δ, *J*_CP_ = 11.9Hz, subst Cp); 77.00 (δ, *J*_CP_ = 93.2Hz, quat Cp); 71.87 (*s*, Cp); 71.85 (δ, *J*_CP_ = 10.3Hz, subst Cp); 69.90 (δ, *J*_CP_ = 8.4Hz, subst Cp); 25.87 (*s*, CH_3_).

**^31^P NMR (δ, ppm, CD_2_Cl_2_)**: δ 41.01.

**HRMS (DCI, CH_4_)**: 471.0638 (100%, calculated for C_26_H_24_FeOPS [*M*] 471.0635).

**M.p.**: 441 K (dec).

**IR (ATR mode, diamond crystal)**: ν_max_(solid)/cm^−1^: 1630 (*s*), 1607 (*s*), 1677 (*w*), 1364 (*m*), 1335 (*m*), 1264 (*s*), 1226 (*m*), 1165 (*s*), 1099 (*s*), 1055 (*m*), 987 (*s*), 863 (*w*), 832 (*m*), 822 (*s*), 760 (*s*), 7478 (*m*), 712 (*s*), 698 (*s*), 690 (*s*), 660 (*s*), 640 (*s*), 614 (*sm*), 583 (*m*), 534 (*s*).

## Refinement   

Crystal data, data collection and structure refinement details are summarized in Table 2 ▸. All H atoms attached to C atoms were fixed geometrically and treated as riding with C—H = 0.95 Å (aromatic) or 0.98 Å (meth­yl) with *U*_iso_(H) = 1.2*U*_eq_(CH aromatic) or *U*_iso_(H) = 1.5*U*_eq_(CH_3_). In the final difference-Fourier map, there is a large residual density, 1.43 e Å^−3^ in the vicinity (1.20 Å) of the H24*A* atom of the terminal methyl group; it is roughly located in the (100) plane; no chemically logical explanation could be found to explain this residual density.

## Supplementary Material

Crystal structure: contains datablock(s) I. DOI: 10.1107/S205698902100760X/zl5019sup1.cif

Structure factors: contains datablock(s) I. DOI: 10.1107/S205698902100760X/zl5019Isup2.hkl

CCDC reference: 2099273

Additional supporting information:  crystallographic information; 3D view; checkCIF report

## Figures and Tables

**Table 1:** Hydrogen-bond geometry (Å, °)

*D*—H⋯*A*	*D*—H	H⋯*A*	*D*⋯*A*	*D*—H⋯*A*
C22—H22⋯O1^i^	0.95	2.63	3.548 (4)	164
C112—H112⋯S1^ii^	0.95	2.83	3.576 (3)	136
C116—H116⋯S1	0.95	2.89	3.374 (3)	113
C21—H21⋯S1	0.95	2.87	3.604 (3)	135

Symmetry codes: (i) -x+1, -y+2, -z+1; (ii) x-1, y, z.

**Table 2:** Experimental details

Crystal data	
Chemical formula	[Fe(C_5_H_5_)(C_21_H_18_OPS)]
*M*_r_	470.32
Crystal system, space group	Monoclinic, *P*2_1_/*c*
Temperature (K)	110
*a*, *b*, *c* (Å)	7.3643 (9), 17.909 (2), 16.710 (2)
β (°)	95.230 (4)
*V* (Å^3^)	2194.8 (5)
*Z*	4
Radiation type	Mo *K*α
μ (mm^−1^)	0.87
Crystal size (mm)	0.1 × 0.07 × 0.01

Data collection	
Diffractometer	Bruker APEXII CCD
Absorption correction	Multi-scan (*SADABS*; Krause *et al.*, 2015 ▸)
*T*_min_, *T*_max_	0.673, 0.730
No. of measured, independent and observed [*I* > 2σ(*I*)] reflections	43009, 5657, 3907
*R*_int_	0.113
(sin θ/λ)_max_ (Å^−1^)	0.688

Refinement	
*R*[*F* ^2^ > 2σ(*F* ^2^)], *wR*(*F* ^2^), *S*	0.053, 0.144, 1.03
No. of reflections	5657
No. of parameters	272
H-atom treatment	H-atom parameters constrained
Δρ_max_, Δρ_min_ (e Å^−3^)	1.43, −0.50

Computer programs: *APEX2* and *SAINT* (Bruker, 2015 ▸), *SHELXT* (Sheldrick, 2015*a* ▸), *SHELXL2018* (Sheldrick, 2015*b* ▸), *ORTEPIII* (Burnett & Johnson, 1996 ▸); *ORTEP-3 for Windows* (Farrugia, 2012 ▸) and *Mercury* (Macrae *et al.*, 2020 ▸).

## supplementary crystallographic information

### Crystal data

**Table:**

[Fe(C_5_H_5_)(C_21_H_18_OPS)]	*F*(000) = 976
*M**_r_* = 470.32	*D*_x_ = 1.425 Mg m^−^^3^
Monoclinic, *P*2_1_/*c*	Mo *K*α radiation, λ = 0.71073 Å
*a* = 7.3643 (9) Å	Cell parameters from 4518 reflections
*b* = 17.909 (2) Å	θ = 2.7–24.2°
*c* = 16.710 (2) Å	µ = 0.87 mm^−^^1^
β = 95.230 (4)°	*T* = 110 K
*V* = 2194.8 (5) Å^3^	Platelet, orange yellow
*Z* = 4	0.1 × 0.07 × 0.01 mm

### Data collection

**Table:**

Bruker APEXII CCD diffractometer	5657 independent reflections
Radiation source: micro-focus sealed tube	3907 reflections with *I* > 2σ(*I*)
Graphite monochromator	*R*_int_ = 0.113
φ and ω scans	θ_max_ = 29.3°, θ_min_ = 3.7°
Absorption correction: multi-scan (SADABS; Krause *et al.*, 2015)	*h* = −10→10
*T*_min_ = 0.673, *T*_max_ = 0.730	*k* = −24→24
43009 measured reflections	*l* = −22→20

### Refinement

**Table:**

Refinement on *F*^2^	Primary atom site location: structure-invariant direct methods
Least-squares matrix: full	Secondary atom site location: difference Fourier map
*R*[*F*^2^ > 2σ(*F*^2^)] = 0.053	Hydrogen site location: inferred from neighbouring sites
*wR*(*F*^2^) = 0.144	H-atom parameters constrained
*S* = 1.03	*w* = 1/[σ^2^(*F*_o_^2^) + (0.0664*P*)^2^ + 1.8414*P*] where *P* = (*F*_o_^2^ + 2*F*_c_^2^)/3
5657 reflections	(Δ/σ)_max_ = 0.001
272 parameters	Δρ_max_ = 1.43 e Å^−^^3^
0 restraints	Δρ_min_ = −0.50 e Å^−^^3^

### Special details

**Table:**

Geometry. All esds (except the esd in the dihedral angle between two l.s. planes) are estimated using the full covariance matrix. The cell esds are taken into account individually in the estimation of esds in distances, angles and torsion angles; correlations between esds in cell parameters are only used when they are defined by crystal symmetry. An approximate (isotropic) treatment of cell esds is used for estimating esds involving l.s. planes.

### Fractional atomic coordinates and isotropic or equivalent isotropic displacement parameters (Å^2^)

**Table:**

	*x*	*y*	*z*	*U*_iso_*/*U*_eq_	
Fe1	0.39674 (5)	0.81999 (2)	0.74273 (2)	0.01578 (14)	
S1	0.61309 (10)	0.63480 (5)	0.64301 (5)	0.0248 (2)	
P1	0.35129 (10)	0.65181 (4)	0.64545 (5)	0.01527 (18)	
O1	0.7131 (3)	0.95064 (13)	0.44772 (14)	0.0292 (5)	
C1	0.2847 (4)	0.74695 (16)	0.65962 (17)	0.0147 (6)	
C2	0.3485 (4)	0.81389 (16)	0.62101 (17)	0.0151 (6)	
C3	0.2527 (4)	0.87556 (17)	0.65055 (18)	0.0183 (6)	
H3	0.267892	0.926260	0.635831	0.022*	
C4	0.1317 (4)	0.84957 (18)	0.70518 (19)	0.0196 (6)	
H4	0.051683	0.879705	0.732889	0.024*	
C5	0.1495 (4)	0.77100 (17)	0.71171 (18)	0.0181 (6)	
H5	0.084008	0.739506	0.744651	0.022*	
C6	0.6382 (4)	0.8733 (2)	0.7754 (2)	0.0296 (8)	
H6	0.699427	0.906469	0.742541	0.036*	
C7	0.6625 (5)	0.7957 (2)	0.7792 (2)	0.0344 (9)	
H7	0.742647	0.767350	0.749659	0.041*	
C8	0.5462 (5)	0.7670 (2)	0.8349 (2)	0.0336 (9)	
H8	0.534168	0.716032	0.849153	0.040*	
C9	0.4507 (5)	0.8281 (2)	0.86554 (19)	0.0287 (8)	
H9	0.363864	0.825418	0.904144	0.034*	
C10	0.5090 (4)	0.89381 (19)	0.8279 (2)	0.0259 (7)	
H10	0.467609	0.943108	0.836689	0.031*	
C21	0.4977 (4)	0.81952 (17)	0.56951 (17)	0.0182 (6)	
H21	0.573671	0.777207	0.564631	0.022*	
C22	0.5325 (4)	0.88155 (17)	0.52879 (17)	0.0192 (6)	
H22	0.446573	0.921101	0.528844	0.023*	
C23	0.6929 (4)	0.89337 (17)	0.48403 (18)	0.0202 (6)	
C24	0.8396 (5)	0.8344 (2)	0.4856 (2)	0.0289 (8)	
H24A	0.918833	0.844327	0.442775	0.043*	
H24B	0.783093	0.785139	0.477308	0.043*	
H24C	0.912153	0.835465	0.537758	0.043*	
C111	0.2541 (4)	0.60087 (16)	0.72528 (17)	0.0157 (6)	
C112	0.0667 (4)	0.58941 (18)	0.72292 (18)	0.0207 (7)	
H112	−0.010291	0.605709	0.677569	0.025*	
C113	−0.0085 (4)	0.55449 (18)	0.78603 (19)	0.0246 (7)	
H113	−0.136781	0.547765	0.784222	0.029*	
C114	0.1032 (5)	0.52926 (19)	0.8520 (2)	0.0265 (7)	
H114	0.051826	0.505580	0.895551	0.032*	
C115	0.2907 (5)	0.53900 (19)	0.8537 (2)	0.0267 (7)	
H115	0.367996	0.520842	0.898038	0.032*	
C116	0.3662 (4)	0.57508 (17)	0.79095 (18)	0.0202 (6)	
H116	0.494365	0.582127	0.792935	0.024*	
C121	0.2198 (4)	0.62196 (17)	0.55413 (17)	0.0172 (6)	
C122	0.0639 (5)	0.65958 (19)	0.5239 (2)	0.0258 (7)	
H122	0.025180	0.702742	0.550738	0.031*	
C123	−0.0366 (5)	0.63428 (19)	0.4543 (2)	0.0294 (8)	
H123	−0.143308	0.660150	0.433628	0.035*	
C124	0.0204 (5)	0.57107 (19)	0.41551 (19)	0.0257 (7)	
H124	−0.046804	0.553753	0.367907	0.031*	
C125	0.1738 (5)	0.53371 (18)	0.4459 (2)	0.0253 (7)	
H125	0.211159	0.490163	0.419431	0.030*	
C126	0.2746 (4)	0.55824 (17)	0.51434 (19)	0.0211 (7)	
H126	0.381137	0.531932	0.534462	0.025*	

### Atomic displacement parameters (Å^2^)

**Table:**

	*U*^11^	*U*^22^	*U*^33^	*U*^12^	*U*^13^	*U*^23^
Fe1	0.0149 (2)	0.0185 (2)	0.0138 (2)	0.00177 (17)	0.00064 (16)	−0.00037 (17)
S1	0.0137 (4)	0.0228 (4)	0.0384 (5)	0.0056 (3)	0.0057 (3)	0.0026 (3)
P1	0.0126 (4)	0.0148 (4)	0.0185 (4)	0.0030 (3)	0.0017 (3)	0.0020 (3)
O1	0.0346 (13)	0.0265 (13)	0.0273 (13)	−0.0097 (11)	0.0069 (10)	0.0070 (10)
C1	0.0107 (13)	0.0166 (15)	0.0165 (14)	0.0022 (11)	0.0000 (11)	0.0008 (11)
C2	0.0122 (13)	0.0180 (15)	0.0145 (14)	0.0019 (11)	−0.0022 (11)	0.0008 (11)
C3	0.0205 (15)	0.0158 (15)	0.0179 (15)	0.0014 (12)	−0.0017 (12)	0.0000 (12)
C4	0.0136 (14)	0.0210 (16)	0.0243 (16)	0.0060 (12)	0.0023 (12)	−0.0021 (13)
C5	0.0092 (13)	0.0238 (17)	0.0217 (15)	0.0022 (12)	0.0033 (11)	0.0012 (12)
C6	0.0207 (17)	0.043 (2)	0.0235 (17)	−0.0084 (15)	−0.0076 (13)	0.0031 (15)
C7	0.0179 (16)	0.055 (3)	0.0286 (19)	0.0117 (16)	−0.0066 (14)	−0.0145 (17)
C8	0.043 (2)	0.0265 (19)	0.0271 (18)	−0.0002 (16)	−0.0214 (16)	0.0042 (15)
C9	0.0310 (18)	0.040 (2)	0.0150 (16)	−0.0072 (16)	0.0017 (13)	−0.0031 (14)
C10	0.0279 (17)	0.0236 (18)	0.0247 (17)	−0.0024 (14)	−0.0059 (13)	−0.0083 (14)
C21	0.0204 (15)	0.0175 (15)	0.0164 (15)	0.0020 (12)	0.0003 (12)	−0.0009 (12)
C22	0.0209 (15)	0.0192 (16)	0.0174 (15)	0.0015 (12)	0.0014 (12)	−0.0006 (12)
C23	0.0218 (16)	0.0196 (16)	0.0188 (15)	0.0001 (13)	−0.0008 (12)	−0.0028 (12)
C24	0.0243 (17)	0.030 (2)	0.0331 (19)	0.0044 (14)	0.0069 (14)	0.0034 (15)
C111	0.0158 (14)	0.0145 (14)	0.0168 (14)	0.0003 (11)	0.0008 (11)	0.0007 (11)
C112	0.0160 (14)	0.0256 (17)	0.0196 (15)	−0.0015 (13)	−0.0038 (12)	0.0041 (13)
C113	0.0203 (16)	0.0262 (18)	0.0273 (18)	−0.0057 (13)	0.0024 (13)	0.0008 (14)
C114	0.0316 (18)	0.0254 (18)	0.0229 (17)	−0.0034 (14)	0.0046 (14)	0.0056 (14)
C115	0.0310 (18)	0.0242 (18)	0.0237 (17)	0.0003 (14)	−0.0049 (14)	0.0095 (14)
C116	0.0187 (15)	0.0168 (16)	0.0241 (16)	0.0009 (12)	−0.0030 (12)	0.0018 (12)
C121	0.0188 (14)	0.0190 (15)	0.0143 (14)	0.0019 (12)	0.0037 (11)	0.0008 (12)
C122	0.0301 (18)	0.0209 (17)	0.0254 (17)	0.0101 (14)	−0.0032 (14)	−0.0046 (13)
C123	0.036 (2)	0.0261 (18)	0.0241 (17)	0.0089 (15)	−0.0073 (14)	−0.0014 (14)
C124	0.0356 (19)	0.0241 (17)	0.0172 (16)	−0.0054 (15)	0.0020 (13)	−0.0005 (13)
C125	0.0314 (18)	0.0191 (16)	0.0271 (18)	−0.0006 (14)	0.0112 (14)	−0.0053 (13)
C126	0.0215 (15)	0.0183 (16)	0.0245 (16)	0.0023 (13)	0.0072 (12)	−0.0015 (13)

### Geometric parameters (Å, º)

**Table:**

Fe1—C1	2.028 (3)	C9—H9	0.9500
Fe1—C2	2.035 (3)	C10—H10	0.9500
Fe1—C7	2.043 (3)	C21—C22	1.340 (4)
Fe1—C8	2.043 (3)	C21—H21	0.9500
Fe1—C5	2.045 (3)	C22—C23	1.470 (4)
Fe1—C3	2.047 (3)	C22—H22	0.9500
Fe1—C6	2.048 (3)	C23—C24	1.509 (4)
Fe1—C10	2.059 (3)	C24—H24A	0.9800
Fe1—C9	2.060 (3)	C24—H24B	0.9800
Fe1—C4	2.064 (3)	C24—H24C	0.9800
S1—P1	1.9560 (11)	C111—C116	1.391 (4)
P1—C1	1.795 (3)	C111—C112	1.392 (4)
P1—C121	1.812 (3)	C112—C113	1.384 (4)
P1—C111	1.816 (3)	C112—H112	0.9500
O1—C23	1.208 (4)	C113—C114	1.390 (5)
C1—C5	1.446 (4)	C113—H113	0.9500
C1—C2	1.459 (4)	C114—C115	1.390 (5)
C2—C3	1.423 (4)	C114—H114	0.9500
C2—C21	1.459 (4)	C115—C116	1.389 (4)
C3—C4	1.412 (4)	C115—H115	0.9500
C3—H3	0.9500	C116—H116	0.9500
C4—C5	1.417 (4)	C121—C122	1.386 (4)
C4—H4	0.9500	C121—C126	1.399 (4)
C5—H5	0.9500	C122—C123	1.396 (4)
C6—C10	1.401 (5)	C122—H122	0.9500
C6—C7	1.401 (5)	C123—C124	1.389 (5)
C6—H6	0.9500	C123—H123	0.9500
C7—C8	1.417 (6)	C124—C125	1.370 (5)
C7—H7	0.9500	C124—H124	0.9500
C8—C9	1.421 (5)	C125—C126	1.377 (5)
C8—H8	0.9500	C125—H125	0.9500
C9—C10	1.420 (5)	C126—H126	0.9500
			
C1—Fe1—C2	42.10 (11)	C10—C6—C7	108.8 (3)
C1—Fe1—C7	112.69 (13)	C10—C6—Fe1	70.49 (19)
C2—Fe1—C7	111.19 (13)	C7—C6—Fe1	69.8 (2)
C1—Fe1—C8	111.98 (13)	C10—C6—H6	125.6
C2—Fe1—C8	139.56 (14)	C7—C6—H6	125.6
C7—Fe1—C8	40.60 (16)	Fe1—C6—H6	125.7
C1—Fe1—C5	41.60 (11)	C6—C7—C8	107.9 (3)
C2—Fe1—C5	69.73 (12)	C6—C7—Fe1	70.16 (19)
C7—Fe1—C5	142.26 (15)	C8—C7—Fe1	69.70 (19)
C8—Fe1—C5	113.44 (14)	C6—C7—H7	126.1
C1—Fe1—C3	69.37 (12)	C8—C7—H7	126.1
C2—Fe1—C3	40.80 (11)	Fe1—C7—H7	125.7
C7—Fe1—C3	138.11 (14)	C7—C8—C9	107.9 (3)
C8—Fe1—C3	178.29 (14)	C7—C8—Fe1	69.70 (19)
C5—Fe1—C3	68.27 (12)	C9—C8—Fe1	70.38 (19)
C1—Fe1—C6	140.55 (13)	C7—C8—H8	126.1
C2—Fe1—C6	111.05 (13)	C9—C8—H8	126.1
C7—Fe1—C6	40.06 (15)	Fe1—C8—H8	125.4
C8—Fe1—C6	67.70 (15)	C10—C9—C8	107.4 (3)
C5—Fe1—C6	177.42 (14)	C10—C9—Fe1	69.82 (18)
C3—Fe1—C6	110.59 (14)	C8—C9—Fe1	69.10 (18)
C1—Fe1—C10	179.48 (13)	C10—C9—H9	126.3
C2—Fe1—C10	138.35 (13)	C8—C9—H9	126.3
C7—Fe1—C10	67.51 (14)	Fe1—C9—H9	126.3
C8—Fe1—C10	67.83 (14)	C6—C10—C9	108.1 (3)
C5—Fe1—C10	137.97 (13)	C6—C10—Fe1	69.62 (19)
C3—Fe1—C10	110.83 (13)	C9—C10—Fe1	69.85 (18)
C6—Fe1—C10	39.90 (14)	C6—C10—H10	126.0
C1—Fe1—C9	139.22 (13)	C9—C10—H10	126.0
C2—Fe1—C9	178.56 (13)	Fe1—C10—H10	126.1
C7—Fe1—C9	68.00 (14)	C22—C21—C2	123.1 (3)
C8—Fe1—C9	40.52 (15)	C22—C21—H21	118.4
C5—Fe1—C9	111.67 (13)	C2—C21—H21	118.4
C3—Fe1—C9	139.05 (13)	C21—C22—C23	125.3 (3)
C6—Fe1—C9	67.54 (14)	C21—C22—H22	117.3
C10—Fe1—C9	40.33 (14)	C23—C22—H22	117.3
C1—Fe1—C4	69.08 (12)	O1—C23—C22	121.3 (3)
C2—Fe1—C4	68.66 (12)	O1—C23—C24	118.8 (3)
C7—Fe1—C4	177.37 (15)	C22—C23—C24	119.8 (3)
C8—Fe1—C4	141.06 (15)	C23—C24—H24A	109.5
C5—Fe1—C4	40.33 (12)	C23—C24—H24B	109.5
C3—Fe1—C4	40.17 (12)	H24A—C24—H24B	109.5
C6—Fe1—C4	137.36 (14)	C23—C24—H24C	109.5
C10—Fe1—C4	110.75 (13)	H24A—C24—H24C	109.5
C9—Fe1—C4	112.09 (13)	H24B—C24—H24C	109.5
C1—P1—C121	105.06 (14)	C116—C111—C112	119.3 (3)
C1—P1—C111	104.43 (13)	C116—C111—P1	120.1 (2)
C121—P1—C111	104.73 (14)	C112—C111—P1	120.6 (2)
C1—P1—S1	115.62 (10)	C113—C112—C111	120.6 (3)
C121—P1—S1	112.86 (10)	C113—C112—H112	119.7
C111—P1—S1	113.10 (10)	C111—C112—H112	119.7
C5—C1—C2	106.8 (2)	C112—C113—C114	120.2 (3)
C5—C1—P1	125.0 (2)	C112—C113—H113	119.9
C2—C1—P1	128.2 (2)	C114—C113—H113	119.9
C5—C1—Fe1	69.82 (17)	C113—C114—C115	119.4 (3)
C2—C1—Fe1	69.23 (16)	C113—C114—H114	120.3
P1—C1—Fe1	127.14 (15)	C115—C114—H114	120.3
C3—C2—C21	125.1 (3)	C116—C115—C114	120.6 (3)
C3—C2—C1	107.1 (2)	C116—C115—H115	119.7
C21—C2—C1	127.4 (3)	C114—C115—H115	119.7
C3—C2—Fe1	70.03 (16)	C115—C116—C111	120.0 (3)
C21—C2—Fe1	120.9 (2)	C115—C116—H116	120.0
C1—C2—Fe1	68.67 (16)	C111—C116—H116	120.0
C4—C3—C2	109.3 (3)	C122—C121—C126	119.3 (3)
C4—C3—Fe1	70.58 (17)	C122—C121—P1	121.6 (2)
C2—C3—Fe1	69.17 (16)	C126—C121—P1	119.1 (2)
C4—C3—H3	125.3	C121—C122—C123	120.3 (3)
C2—C3—H3	125.3	C121—C122—H122	119.9
Fe1—C3—H3	126.5	C123—C122—H122	119.9
C3—C4—C5	108.5 (3)	C124—C123—C122	119.6 (3)
C3—C4—Fe1	69.25 (17)	C124—C123—H123	120.2
C5—C4—Fe1	69.09 (16)	C122—C123—H123	120.2
C3—C4—H4	125.7	C125—C124—C123	119.9 (3)
C5—C4—H4	125.7	C125—C124—H124	120.0
Fe1—C4—H4	127.5	C123—C124—H124	120.0
C4—C5—C1	108.3 (3)	C124—C125—C126	121.1 (3)
C4—C5—Fe1	70.58 (17)	C124—C125—H125	119.5
C1—C5—Fe1	68.58 (16)	C126—C125—H125	119.5
C4—C5—H5	125.9	C125—C126—C121	119.9 (3)
C1—C5—H5	125.9	C125—C126—H126	120.1
Fe1—C5—H5	126.5	C121—C126—H126	120.1

### Hydrogen-bond geometry (Å, º)

**Table:**

*D*—H···*A*	*D*—H	H···*A*	*D*···*A*	*D*—H···*A*
C22—H22···O1^i^	0.95	2.63	3.548 (4)	164
C112—H112···S1^ii^	0.95	2.83	3.576 (3)	136
C116—H116···S1	0.95	2.89	3.374 (3)	113
C21—H21···S1	0.95	2.87	3.604 (3)	135

Symmetry codes: (i) −*x*+1, −*y*+2, −*z*+1; (ii) *x*−1, *y*, *z*.
